# Cat and Mouse Based Optimizer: A New Nature-Inspired Optimization Algorithm

**DOI:** 10.3390/s21155214

**Published:** 2021-07-31

**Authors:** Mohammad Dehghani, Štěpán Hubálovský, Pavel Trojovský

**Affiliations:** 1 Department of Mathematics, Faculty of Science, University of Hradec Králové, 500 03 Hradec Králové, Czech Republic; mohammad.dehghani@uhk.cz; 2 Department of Applied Cybernetics, Faculty of Science, University of Hradec Králové, 500 03 Hradec Králové, Czech Republic; stepan.hubalovsky@uhk.cz

**Keywords:** optimization, population-based, stochastic, cat and mouse, optimization problem

## Abstract

Numerous optimization problems designed in different branches of science and the real world must be solved using appropriate techniques. Population-based optimization algorithms are some of the most important and practical techniques for solving optimization problems. In this paper, a new optimization algorithm called the Cat and Mouse-Based Optimizer (CMBO) is presented that mimics the natural behavior between cats and mice. In the proposed CMBO, the movement of cats towards mice as well as the escape of mice towards havens is simulated. Mathematical modeling and formulation of the proposed CMBO for implementation on optimization problems are presented. The performance of the CMBO is evaluated on a standard set of objective functions of three different types including unimodal, high-dimensional multimodal, and fixed-dimensional multimodal. The results of optimization of objective functions show that the proposed CMBO has a good ability to solve various optimization problems. Moreover, the optimization results obtained from the CMBO are compared with the performance of nine other well-known algorithms including Genetic Algorithm (GA), Particle Swarm Optimization (PSO), Gravitational Search Algorithm (GSA), Teaching-Learning-Based Optimization (TLBO), Grey Wolf Optimizer (GWO), Whale Optimization Algorithm (WOA), Marine Predators Algorithm (MPA), Tunicate Swarm Algorithm (TSA), and Teamwork Optimization Algorithm (TOA). The performance analysis of the proposed CMBO against the compared algorithms shows that CMBO is much more competitive than other algorithms by providing more suitable quasi-optimal solutions that are closer to the global optimal.

## 1. Introduction

### 1.1. Motivation

Optimization is the adjustment and modification of the inputs and properties of a device, a mathematical process, or an experimental experiment in order to obtain the best output or result. Each optimization problem has three main parts: decision variables, constraints, and objective functions [1]. Decision variables should be adjusted and quantified in such a way that the objective function of the problem is optimized according to the constraints. In fact, there are several solutions to an optimization problem where finding the best solution is the main challenge in optimizing the objective function [2].

### 1.2. Literature Review

Optimization problem solving methods from the general point of view are grouped into two categories: (i) deterministic methods and (ii) stochastic methods [3].

Deterministic methods also are grouped into two categories: (i) gradient-based and (ii) non-gradient-based methods. Gradient-based methods are valid and easy to use for simple cost functions. Many complex problems can be transformed into functions with a little modification that can be solved using these methods. However, with increasing dimensions of the problem, as well as in nonlinear search spaces, these methods are simply stuck in local optimal solutions and are not able to provide a suitable solution. Non-gradient-based methods use condition and objective function evaluation to converge to the solution. However, the main disadvantage of these methods is that they are very dependent on the initial conditions and their implementation requires high experience and knowledge of mathematics [4].

#### Population-Based Optimization Algorithms

Population-based optimization algorithms is one of the most widely used methods for solving optimization problems, which belongs to the group of stochastic methods [5]. Population-based optimization algorithms without the need to derivative and gradients information and based on search operators and collective intelligence are able to provide appropriate solutions to optimization problems by randomly scanning the search space [6]. Optimization algorithms have been developed based on the ideation of various natural phenomena, the natural behaviors of animals and living organisms, the laws of physics, the genetic sciences, the rules of games, and other processes that have the potential to evolve.

Genetic Algorithm (GA) is one of the oldest and most widely used optimization methods in solving optimization problems, which is developed based on Darwin’s theory of evolution and reproduction process simulation. In GA, three operators of selection, crossover, and mutation is applied to model reproduction according to the law of survival of the fittest and the evolution of the offspring [7]. The advantages of GA are that it has simple and understandable concepts, but having control parameters that must be well adjusted and also time-consuming implementation are the most important disadvantages of this algorithm.

Particle Swarm Optimization (PSO) is another widely used algorithm which is based on the imitation of bird and fish swarm motion. In PSO, the strategy of moving particles and updating search agents is based on the best personal experience of each particle and the global experience of the entire population [8]. The simplicity of mathematical equations and their easy implementation are the main advantages of PSO. The main disadvantages of PSO algorithm are falling into the trap of local optimal, reduced population diversity, and low convergence speed.

Gravitational Search algorithm (GSA) is a physics-based algorithm which is inspired by gravitational force and Newton’s laws of motion. In GSA, the gravitational force is modeled between different objects that are actually members of the algorithm population and are at different distances from each other. The acceleration, velocity, and displacement of objects are then updated according to Newton’s laws of motion [9]. Fast convergence in simple problems, easy implementation, and low computational cost are the main advantages of GSA. Among the disadvantages of the GSA are slow convergence, time-consumption, and the tendency to become trapped in local optimal solutions.

Teaching Learning-based Optimization (TLBO) is a population-based technique which is developed based on modeling behaviors and interactions between students and the teacher in the classroom. TLBO updates the algorithm population in two phases of teacher and learner. In the teacher phase, the educational behavior of the teacher, who is the best member of the population, towards the students is modeled. In the learner phase, students share their knowledge and information with each other [10]. Good global search, simplicity, and no requirement to control parameters are the main advantages of TLBO. Disadvantages of TLBO is that consumes lot of memory space and involves lot of iterations so is a time-consuming method.

Grey Wolf Optimizer (GWO) is inspired by social life and hunting strategy of the gray wolves in nature. In GWO, the hierarchical behavior of leadership in gray wolves is modeled using four types of wolves: alpha, beta, delta, and omega. The hunting strategy is also simulated in three stages including search for prey, encircling prey, and attacking prey [11]. Easily implementation, fewer storage and computational requirements are the main advantages of GWO. Slow convergence, low solving precision, having controller parameters, and bad local searching ability are the main disadvantages of GWO.

Whale Optimization Algorithm (WOA) is a nature-inspired algorithm which is developed based on social behavior of humpback whales and bubble-net hunting strategy. WOA have three operators to simulate the search for prey, encircling prey, and bubble-net foraging behavior of humpback whales [12]. Appropriate balance between exploration and exploitation is the main advantage of WOA. The main disadvantages of WOA are slow convergence speed, weak exploring search space, and easy falling into local optimal.

Marine Predators Algorithm (MPA) is introduced based on the movement strategies that marine predators use when trapping their prey in the oceans. MPA performance is simulated based on the behavior and strategy of search and pursuit of marine predators due to the different speeds of predators and prey in three phases. Phase (i): When the prey moves faster than the predator; Phase (ii): When the prey and the predator move at almost the same speed; and Phase (iii): When the predator is moving faster than the prey [13]. Good global search and fast convergence are the main advantages of MPA. The main disadvantages of MPA are lack of escaping from the local optimization, the inability to produce a diverse initial population with high productivity, and lack of broadly and widely exploration of the search space.

Tunicate Swarm Algorithm (TSA) is a bio-inspired method which is introduced based on simulation of jet propulsion and swarm behaviors of tunicates during the navigation and foraging process. In TSA the jet propulsion behavior is simulated considering three conditions including movement towards the position of best search agent, avoid the conflicts between search agents, and remains close to the best search agent [14]. Good global search and appropriate balance between exploration and exploitation are the main advantages of TSA. Low convergence rate and weakness in local search are the main disadvantages of TSA.

Teamwork Optimization Algorithm (TOA) is a population-based approach which is developed based on mathematical modeling of relationships and interactions between team members in doing a teamwork to achieve the goal of that team. In TOA, team members are updated on each iteration in three phases: supervisor guidance, information sharing, and individual activity [15]. Although TOA has advantages such as not requiring any parameter controlling, good global search, having appropriate balance between exploration and exploitation, and fast convergence, fall to local optimal solutions in solving high-dimensional multimodal problems is the most important drawback of this algorithm.

In addition, several well-known optimization algorithms in the recent literature are represented in Table 1.

### 1.3. Research Gap and Question

Every optimization problem has a basic solution called global optimal solution. The important thing about optimization algorithms is that there is no guarantee that the solutions obtained from these methods necessarily be global optimal solution. For this reason, the solutions that are obtained using optimization algorithms for optimization problems are called quasi-optimal solutions [51].

At best, the quasi-optimal solution is equal to the global optimal solution; otherwise, it must be close to it. Therefore, in analyzing the performance of several optimization algorithms in solving an optimization problem, the algorithm that is able to provide a quasi-optimal solution closer to the global optimal solution is the superior algorithm for solving that optimization problem. Another point is that the optimization algorithm may work very well in solving the optimization problem, but it will not be able to solve another optimization problem. That is why researchers have developed many optimization algorithms to achieve quasi-optimal solutions that are more appropriate and closer to the global optimal solution.

In order to evaluate the performance of optimization algorithms in achieving quasi-optimal solutions, optimization algorithms are implemented on standard optimization problems as benchmark functions whose optimal solution is already known. The criterion of superiority of optimization algorithms over each other is to provide a solution closer to the global optimal. Therefore, it is always possible to design a new optimization algorithm that provides better performance than existing algorithms in optimizing optimization problems. In this regard, the main research question of this paper is whether it is possible to design a new optimization algorithm that can provide a quasi-optimal solution closer to a global optimal solution.

### 1.4. Contribution and Applications

In this paper, a new stochastic method called Cat and Mouse Optimization Algorithm (CMBO) is introduced to solve various optimization problems and provide suitable quasi-optimal solutions. The contributions proposed by this paper are as follows:

(i) (CMBO is designed based on the simulation of natural interactions between cat and mouse.
(ii) The various steps and theory of the proposed CMBO are described and its mathematical model is presented to use in optimizing objective functions.
(iii) The capability of the CMBO in solving optimization problems has been tested on twenty-three standard objective functions.
(iv) The results obtained from the CMBO are also compared with the performance of nine well-known optimization algorithms.

Optimization algorithms are used in all disciplines and real-world problems where the optimization process or problem is designed and defined. The proposed CMBO can be used to minimize or maximize various objective functions. CMBO can be used in engineering sciences and optimal designs where decision variables must be well selected to optimize device performance. In medical science, data mining, clustering, and in general in any application that faces optimization, the proposed CMBO can be used.

### 1.5. Paper Organization

The rest of this paper is organized in such a way that the proposed CMBO is introduced in Section 2. Simulation studies and evaluation of the CMBO are presented in Section 3. The discussion and analysis of the results is presented in Section 4. Finally, in Section 5, conclusions as well as several suggestions for future studies are provided.

## 2. Cat and Mouse Optimization Algorithm

In this section, the theory of the Cat and Mouse Optimization Algorithm (CMBO) is stated, then its mathematical model is presented in order to use in optimizing various problems.

The CMBO is a population-based algorithm which is designed by inspiration from the natural behaviors of a cat attacks on mouse and mouse escape to the haven. The search agents in the proposed algorithm are divided into two groups of cats and mice that scan the problem search space with random movements. The proposed algorithm updates population members in two phases. In the first phase, the movement of cats towards mice is modeled, and in the second phase, the escape of mice to havens to save its lives is modeled.

From a mathematical point of view, each member of the population is a proposed solution to the problem. In fact, a member of the population specifies values for the problem variables according to its position in the search space. Thus, each member of the population is a vector whose values determine the variables of the problem. The population of the algorithm is determined using a matrix called the population matrix in Equation (1).

(1) $$X={\left[\begin{array}{c} X_{1}\\ \vdots \\ X_{i}\\ \vdots \\ X_{N} \end{array}\right]}_{N\times m}={\left[\begin{array}{ccccc} x_{1,1} & \cdots & x_{1,d} & \cdots & x_{1,m}\\ \vdots & \ddots & \vdots & ⋰ & \vdots \\ x_{i,1}\; & \cdots & x_{i,d} & \cdots & x_{i,m}\\ \vdots & ⋰ & \vdots & \ddots & \vdots \\ x_{N,1} & \cdots & x_{N,d} & \cdots & x_{N,m} \end{array}\right]}_{N\times m},$$

where X is the population matrix of CMBO, $X_{i}$ is the *i*th search agent, $x_{i,d}$ is the value for the *d*th problem variable obtained by the *i*th search agent, N is the number of population members, and m is the number of problem variables.

As mentioned, each member of the population determines the proposed values for the problem variables. Therefore, for each member of the population, a value is specified for the objective function. The values obtained for the objective function are denoted using a vector in Equation (2).

(2) $$F={\left[\begin{array}{c} F_{1}\\ \vdots \\ F_{i}\\ \vdots \\ F_{N} \end{array}\right]}_{N\times 1},$$

where F is the vector of objective function values and $F_{i}$ is the objective function value for the *i*th search agent.

Based on the values obtained for the objective functions, the members of the population are ranked from the best member with the lowest value of the objective function to the worst member of the population with the highest value of the objective function. The sorted population matrix as well as the sorted objective function are determined using Equations (3) and (4).

(3) $$X^{S}={\left[\begin{array}{c} X_{1}^{S}\\ \vdots \\ X_{i}^{S}\\ \vdots \\ X_{N}^{S} \end{array}\right]}_{N\times m}={\left[\begin{array}{ccccc} x_{1,1}^{s} & \cdots & x_{1,d}^{s} & \cdots & x_{1,m}^{s}\\ \vdots & \ddots & \vdots & ⋰ & \vdots \\ x_{i,1}^{s}\; & \cdots & x_{i,d}^{s} & \cdots & x_{i,m}^{s}\\ \vdots & ⋰ & \vdots & \ddots & \vdots \\ x_{N,1}^{s} & \cdots & x_{N,d}^{s} & \cdots & x_{N,m}^{s} \end{array}\right]}_{N\times m},$$

(4) $$F^{S}={\left[\begin{array}{cc} F_{1}^{S} & min\left(F\right)\\ \vdots & \vdots \\ F_{N}^{S} & \max\left(F\right) \end{array}\right]}_{N\times 1},$$

where $X^{S}$ is the sorted population matrix based on objective function value, $X_{i}^{S}$ is the *i*th member of sorted population matrix, $x_{i,d}^{s}$ is the value for the *d*th problem variable obtained by the *i*th search agent of sorted population matrix, and $F^{S}$ is the sorted vector of an objective function.

The population matrix in the proposed CMBO consists of two groups of cats and mice. In the CMBO, it is assumed that half of the population members who provided better values for the objective function constitute the population of mice and the other half of the population members who provided lower values for the objective function constitute the cat population. Based on this concept, the populations of mice and cats are determined in Equations (5) and (6), respectively.

(5) $$M={\left[\begin{array}{c} M_{1}=X_{1}^{S}\\ \vdots \\ M_{i}=X_{i}^{S}\\ \vdots \\ M_{N_{m}}=X_{N_{m}}^{S} \end{array}\right]}_{N_{m}\times m}={\left[\begin{array}{ccccc} x_{1,1}^{s} & \cdots & x_{1,d}^{s} & \cdots & x_{1,m}^{s}\\ \vdots & \ddots & \vdots & ⋰ & \vdots \\ x_{i,1}^{s}\; & \cdots & x_{i,d}^{s} & \cdots & x_{i,m}^{s}\\ \vdots & ⋰ & \vdots & \ddots & \vdots \\ x_{N_{m},1}^{s} & \cdots & x_{N_{m},d}^{s} & \cdots & x_{N_{m},m}^{s} \end{array}\right]}_{N_{m}\times m},$$

(6) $$C={\left[\begin{array}{c} C_{1}=X_{N_{m}+1}^{S}\\ \vdots \\ C_{j}=X_{N_{m}+j}^{S}\\ \vdots \\ C_{N_{c}}=X_{N_{m}+N_{c}}^{S} \end{array}\right]}_{N_{c}\times m}={\left[\begin{array}{ccccc} x_{N_{m}+1,1}^{s} & \cdots & x_{N_{m}+1,d}^{s} & \cdots & x_{N_{m}+1,m}^{s}\\ \vdots & \ddots & \vdots & ⋰ & \vdots \\ x_{N_{m}+j,1}^{s}\; & \cdots & x_{N_{m}+j,d}^{s} & \cdots & x_{N_{m}+j,m}^{s}\\ \vdots & ⋰ & \vdots & \ddots & \vdots \\ x_{N_{m}+N_{c},1}^{s} & \cdots & x_{N_{m}+N_{c},d}^{s} & \cdots & x_{N_{m}+N_{c},m}^{s} \end{array}\right]}_{N_{c}\times m},$$

where M is the population matrix of mice, $N_{m}$ is the number of mice, $M_{i}$ is the *j*th mouse, C is the population matrix of cats, $N_{c}$ is the number of cats, and $C_{j}$ is the *i*th cat.

In order to update the search factors, in the first phase, the change of position of cats is modeled based on the natural behavior of cats and movement towards mice. This phase of the update of the proposed CMBO is mathematically modeled using Equations (7)–(9).

(7) $$C_{j}^{new}:\;c_{j,d}^{new}=c_{j,d}+r\times \left(m_{k,d}-I\times c_{j,d}\right)\;\&\;j=1:N_{c},\;d=1:m,\;k\in 1:N_{m},$$

(8) $$I=round\left(1+rand\right),$$

(9) $$C_{j}=\left\{\begin{array}{c} C_{j}^{new},\;|F_{j}^{c,new}<F_{j}^{c}\\ C_{j},\;|else \end{array}\right.,$$

Here, $C_{j}^{new}$ is the new status of the *j*th cat, $c_{j,d}^{new}$ is the new value for the *d*th problem variable obtained by the *j*th cat, r is a random number in interval $\left[0,1\right]$, $m_{k,d}$ is the *d*th dimension of the *k*th mouse, $F_{j}^{c,new}$ is the objective function value based on new status of the *j*th cat.

In the second phase of the proposed CMBO, the escape of mice to havens is modeled. In CMBO, it is assumed that there is a random haven for each mouse, and mice take refuge in these havens. The position of the havens in the search space is randomly created based on patterning the positions of different members of the algorithm. This phase of updating the position of mice is mathematically modeled using Equations (10)–(12).

(10) $$H_{i}:h_{i,d}=x_{l,d}\;\&\;i=1:N_{m},\;d=1:m,\;l\in 1:N,$$

(11) $$M_{i}^{new}:\;m_{i,d}^{new}=m_{i,d}+r\times \left(h_{i,d}-I\times m_{i,d}\right)\times sign\left(F_{i}^{m}-F_{i}^{H}\right)\;\&\;\;i=1:N_{m},\;d=1:m,$$

(12) $$M_{i}=\left\{\begin{array}{c} M_{i}^{new},\;|F_{i}^{m,new}<F_{i}^{m}\\ M_{i},\;|else \end{array}\right.,$$

Here,$\;H_{i}$ is the haven for the *i*th mouse and $F_{i}^{H}$ is its objective function value. $M_{i}^{new}$ is the new status of the *i*th mouse and $F_{i}^{m,new}$ is its objective function value. 

**Figure 1:**
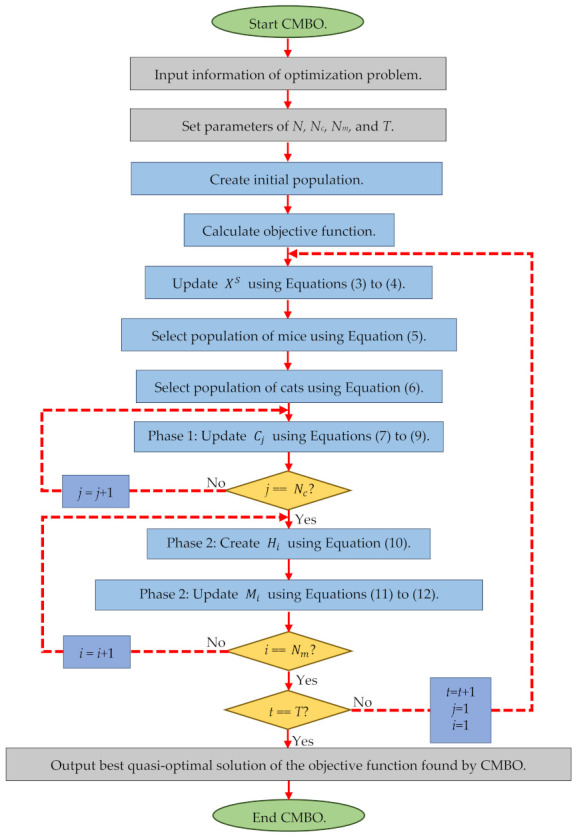
Flowchart of CMBO.

After all members of the algorithm population have been updated, the algorithm enters the next iteration and, based on Equations (5)–(12), the iterations of the algorithm continue until the stop condition is reached. The condition for stopping optimization algorithms can be a certain number of iterations, or by defining an acceptable error between obtained solutions in consecutive iterations. Moreover, the condition for stopping the algorithm may be a certain period of time. Upon completion of the iterations and full implementation of the algorithm on the optimization problem, the CMBO provides the best obtained quasi-optimal solution. Flowcharts of different stages of the proposed CMBO are specified in Figure 1 and its pseudocode is also presented in Algorithm 1.
**Algorithm 1**. Pseudocode of CMBOStart CMBO.
Input problem information: variables, objective function, and constraints.
Set number of search agents (*N*) and iterations (*T*).
Generate an initial population matrix at random.
Evaluate the objective function.

For *t* = 1:*T*


Sort population matrix based on objective function value using Equations (3) and (4).


Select population of mice M using Equation (5).


Select population of cats C using Equation (6).


Phase 1: update status of cats.



For *j* = 1:*N_c_*



Update status of the *j*th cat using Equations (7)–(9).



end


Phase 2: update status of mice.



For *i* = 1:*N_m_*



Create haven for the *i*th mouse using Equation (10).



Update status of the *i*th mouse using Equations (11) and (12).



end

End
Output best quasi-optimal solution obtained with the CMBO.End CMBO

### Step-by-Step Example

In this subsection, a step-by-step example of how to implement the proposed CMBO is provided to explain it in more detail. In this example, CMBO is applied to optimize the sphere function. In this example, it is assumed that the number of problem variables is 2, the number of population members is 10, and the condition of stopping the algorithm is 50 iterations. The mathematical model and information of the sphere function are as follows:

Sphere function:

$$\begin{array}{c} F\left(X\right)=\sum_{d=1}^{m}x_{d}^{2}=F\left(x_{1},x_{2}\right)=x_{1}^{2}+x_{2}^{2}\\ subject\;to:-100\leq x_{1},x_{2}\leq 100 \end{array}$$

Step 1:

In this step, the initial population of feasible solutions is created randomly. The following general formula is used to define the initial random population:

$$X_{i}:x_{d}=x_{lo}+rand\times \left(x_{hi}-x_{lo}\right)\;where\;i=1:N,\;d=1:m$$

For example:

$$\begin{array}{cc} X_{1}: & \\ x_{1}=-100+rand\times \left(100-\left(-100\right)\right):\;x_{1}=69.00641\\ x_{2}=-100+rand\times \left(100-\left(-100\right)\right):\;x_{2}=-74.5553 \end{array}$$

Step 2:

In this step, each member of the population is evaluated in the objective function of the problem. In fact, each member of the population proposes values for the problem variables based on which the objective function can be evaluated.

For example:

$$F_{1}:F\left(X_{1}\right)=F\left(69.00641,-74.5553\right)=10320.38$$

Step 3:

In this step, based on comparing the values obtained for the objective function, the population members are sorted from the best solution (minimum value of the objective function) to the worst solution (maximum value of the objective function). Thus, the sort criterion is the value of the objective function.

Step 4:

In this step, the population of mice (first half of the population with better objective function values) and the population of cats (second half of the population with worse objective function values) are determined according to Equations (5) and (6).

Step 5:

In this step, the position of the cats is updated based on Equations (7)–(9).

Step 6:

In this step, the position of the mice is updated based on Equations (10)–(12).

Step 7:

The third to sixth steps of the algorithm are repeated until the stop condition is met. Finally, after the full implementation of the proposed algorithm on the objective function, the best proposed solution using CMBO is presented for the problem.

The calculations of the different steps of CMBO for the first iteration are presented in Table 2. The final solution for the intended problem after full implementation is also specified in this table.

## 3. Simulation Study and Results

In this section, the efficiency and ability of the proposed CMBO in solving various optimization problems and providing quasi-optimal solutions are evaluated. For this purpose, a standard set consisting of twenty-three objective functions of different types in three groups of unimodal, high-dimensional multimodal, and fixed-dimensional multimodal is applied. Complete information on these functions is provided in Appendix A and Table A1, Table A2 and Table A3.

In order to analyze the quality of the proposed algorithm, the results obtained from the CMBO are compared with nine other optimization algorithms including (i) popular and widely used algorithms: Genetic Algorithm (GA), Particle Swarm Optimization (PSO); (ii) highly cited algorithms: Gravitational Search Algorithm (GSA), Teaching-Learning-Based Optimization (TLBO), Grey Wolf Optimizer (GWO), Whale Optimization Algorithm (WOA); and (iii) recently published algorithms: Tunicate Swarm Algorithm (TSA), Marine Predators Algorithm (MPA), and Teamwork Optimization Algorithm (TOA). The performance results of optimization algorithms are presented using two indicators of average of the best quasi-optimal solutions (ave) and standard deviation of the best quasi-optimal solutions (std). The used values for the parameters of the optimization algorithms are specified in Table 3.

### 3.1. Evaluation of Unimodal Objective Functions

Objective functions F1 to F7 are considered to analyze and evaluate the ability of optimization algorithms to solve and optimize unimodal optimization problems. The results of the implementation of the proposed CMBO as well as nine compared optimization algorithms are presented in Table 4. The proposed algorithm provides the global optimal solution for F6. In addition, CMBO performs very well in the F1, F2, F3, F4, F5, and F7 functions and provides quasi-optimal solutions that are close to the global optimal. Analysis and comparison of the results obtained from the proposed algorithm against the other nine optimization algorithms shows that the CMBO has a higher ability to solve unimodal optimization problems.

### 3.2. Evaluation of High-Dimensional Objective Functions

Six F8 to F13 objective functions of the high-dimensional multi-model functions are selected to evaluate the ability of optimization algorithms to provide optimal quasi-optimal solutions. The results of optimization of these objective functions using the proposed CMBO and nine compared algorithms are presented in Table 5. CMBO provides the global optimal solution for the objective functions of F9 and F11. For F12 and F13 functions, CMBO provides the best performance and provides suitable quasi-optimal solutions. The optimization results show that CMBO obtains very competitive results in majority of the objective functions than other algorithms.

### 3.3. Evaluation of Fixed-Dimensional Objective Functions

F14 to F23 objective functions are selected to evaluate the ability of optimization algorithms to provide suitable solutions for fixed-dimensional multimodal optimization problems. The results of the implementation of optimization algorithms on this type of objective functions are presented in Table 6. CMBO provides good performance in all F14 to F23 objective functions and provides appropriate quasi-optimal solutions for these objective functions. In addition, comparison and analysis of the results show that the proposed algorithm is provided more appropriate solutions in most cases. On the other hand, in functions where CMBO has a similar performance in index “ave” with some algorithms, it is able to solve these optimization problems more effectively with a more appropriate index “std”.

**Figure 2:**
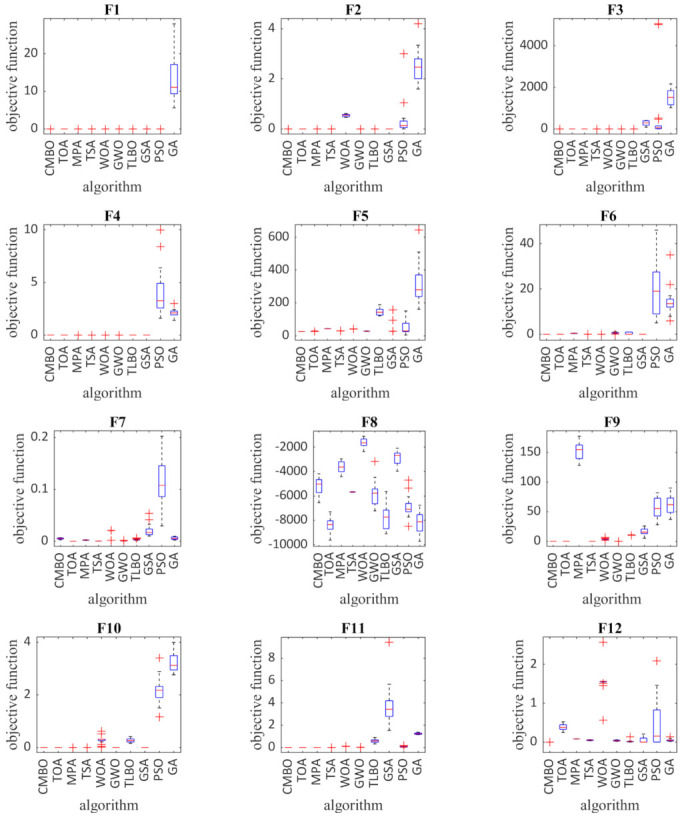

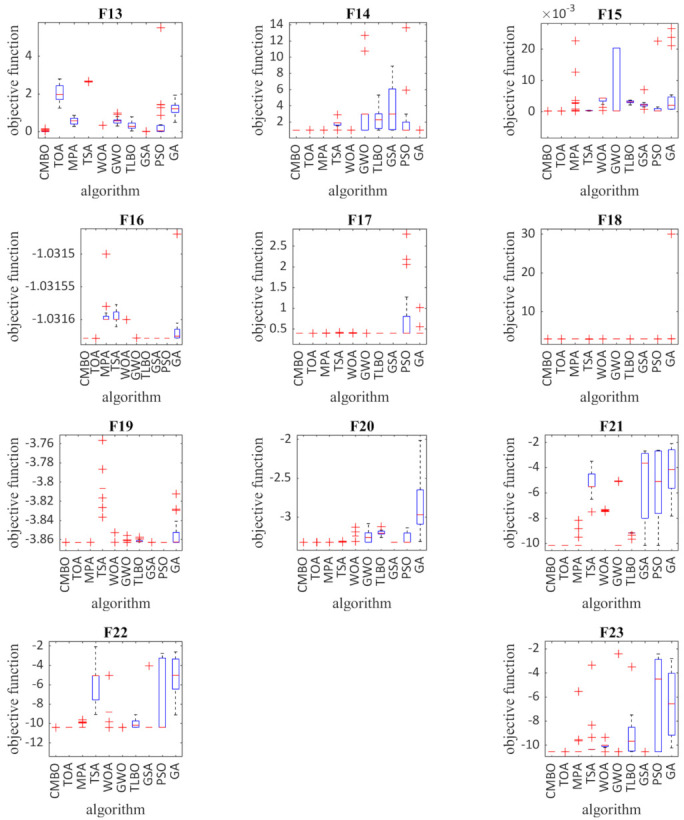
Boxplot of composition objective functions results for different optimization algorithms.

In order to further analyze and visually compare the performance of the optimization algorithms, the boxplot of results for each algorithm and objective function is shown in Figure 2. In Table 4, Table 5 and Table 6, the bold results indicate an algorithm that has performed better in optimizing the specified function.

### 3.4. Statistical Analysis

Presentation and analysis of optimization results using the two indicators of the average of the best results and the standard deviation of the best results provide valuable and useful information about the performance of optimization algorithms. However, even with a very low probability, the superiority of one algorithm over several other algorithms may be coincidental. In this regard, in this subsection, a statistical analysis called Wilcoxon rank-sum test is presented in order to further evaluate and analyze the performance of optimization algorithms as well as the proposed CMBO. The Wilcoxon rank-sum test is one of the nonparametric tests which is used in statistical analysis.

In the Wilcoxon test, a p-value determines whether the considered optimization algorithm is statistically significant or not. If the p-value of the algorithm is less than 0.05, the result is that the algorithm is statistically significant. Table 7 presents the simulation results of statistical analysis using Wilcoxon rank-sum test. What can be concluded from the comparison and analysis of the values presented in this table is that the proposed CMBO has a significant superiority over the compared algorithm in cases where the p-value is less than 0.05. In fact, a p-value indicates whether the proposed CMBO has significant superiority over the compared algorithms. Based on the simulation results, the proposed CMBO has a significant superiority over MPA, WOA, GSA, PSO and GA in optimizing the F1 to F7 unimodal function group. In the second group of objective functions including F8 to F13, CMBO has a significant superiority over TSA, MPA, WOA, GWO, and GSA. The proposed CMBO in optimizing the objective functions of the third group, including F14 to F23, has a significant superiority over all TSA, MPA, WOA, GWO, TLBO, GSA, PSO, GA.

### 3.5. Sensitivity Analysis

In this subsection, the sensitivity analysis of the proposed CMBO with respect to the two parameters of the number of population members of the algorithm and the maximum number of iterations of the algorithm is presented.

**Figure 3:**
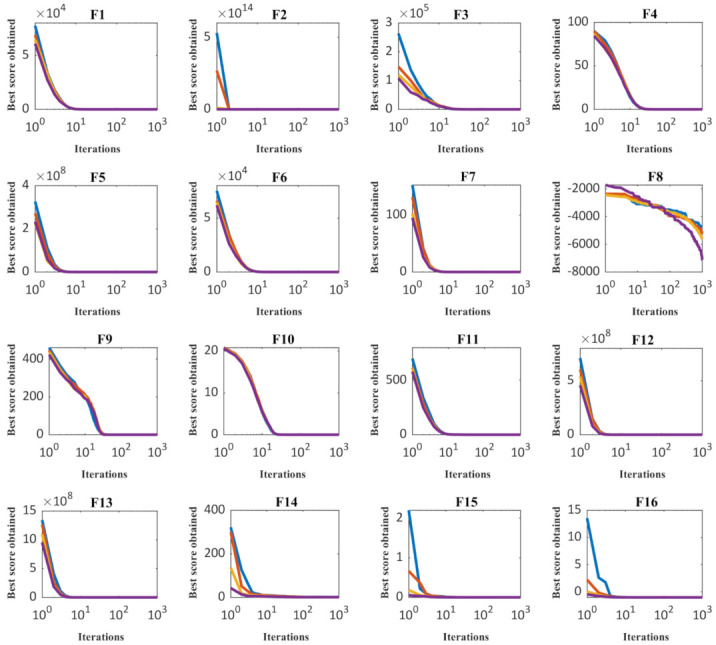

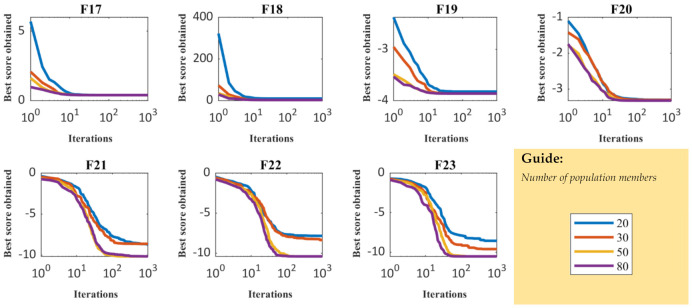
Sensitivity analysis of CMBO for number of population members.

In order to sensitivity analyze of the performance of the CMBO to the number of parameters of population members, it has been implemented on all twenty-three objective functions for different populations with 20, 30, 50, and 80 members. The results of this analysis are presented in Table 8, and also the behavior of convergence curves due to changes in the number of population members is presented in Figure 3. What has been concluded from the simulation results of the sensitivity analysis to the number of population member’s parameter is that as the number of members of the algorithm increases, the proposed CMBO converges to more suitable quasi-optimal solutions and the values of the objective function decrease.

**Figure 4:**
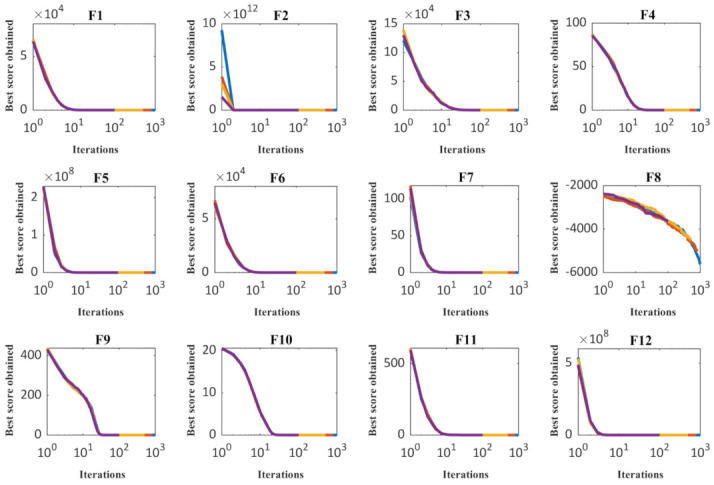

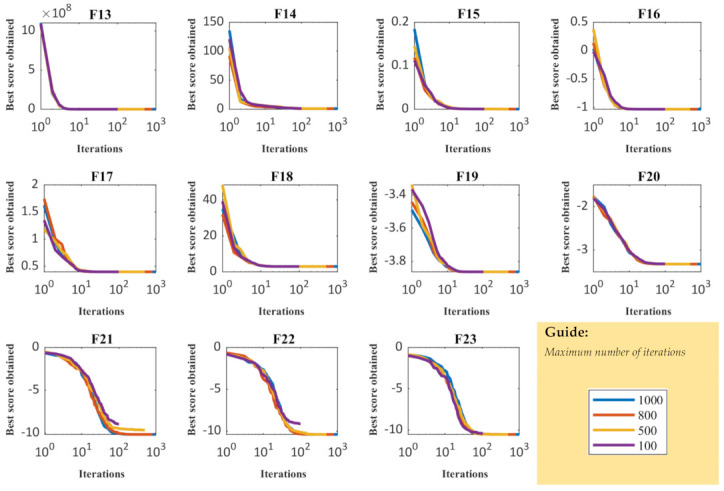
Sensitivity analysis of CMBO for maximum number of iterations.

In order to sensitivity analyze of the performance of the CMBO to the maximum number of iterations parameter, the proposed algorithm has been run independently on all twenty-three objective functions for the maximum number of iterations equal to 100, 500, 800, and 1000. Table 9 presents the evaluation results of this analysis and the behavior of convergence curves under the influence of changes in the maximum number of iterations is presented in Figure 4. The simulation results of the sensitivity analysis of the proposed CMBO with respect to the maximum number of iterations parameter indicate that increasing the maximum number of iterations has led the CMBO to converge to solutions closer to the global optimal.

## 4. Discussion

Exploitation and exploration are two important criteria that play a valuable role in evaluating and determining the quality of optimization algorithms. Optimization algorithms must have a favorable situation in these two criteria in order to be able to have acceptable performance in solving optimization problems.

The concept of exploitation means the ability of optimization algorithms to achieve a suitable quasi-optimal solution that is close to the global optimal. In fact, an optimization algorithm must provide a suitable quasi-optimal solution to an optimization problem after fully implemented. Therefore, in analyzing the effectiveness of several optimization algorithms in solving an optimization problem, the algorithm that suggests a better quasi-optimal solution for that problem has a higher quality in the criterion of exploitation. This criterion is especially important for optimization problems that have only one main solution. The F1 to F7 objective functions, which are selected as unimodal functions, have only one main optimal solution and no optimal local areas. These types of functions are suitable for evaluating the exploitation criterion because of this feature. The results of optimization of these objective functions using the proposed CMBO as well as nine compared algorithms are presented in Table 4. The analysis of these results indicates that the CMBO with high exploitation capability has been able to provide suitable quasi-optimal solutions for F1 to F7 functions, which have a much higher quality than similar algorithms. Therefore, the CMBO is in a much better position than the nine compared algorithms in the exploitation criterion.

The concept of exploration means the ability of optimization algorithms to accurately and appropriately scan the search space of an optimization problem. In fact, optimization algorithms must be able to search different areas of the search space in order to achieve solutions closer to the global optimization. Therefore, in analyzing the performance of several optimization algorithms, an algorithm has a higher quality in the exploration index that provides a more suitable quasi-optimal solution by accurately scanning the search space. This indicator is especially important in optimization problems that have local optimal solutions in addition to the main optimal solution. The F8 to F13 high-dimensional multimodal functions and the F14 to F23 fixed-dimensional multimodal functions have optimal local solutions in addition to basic optimal solution; therefore, these functions are suitable for evaluating the exploration power of optimization algorithms. The optimization results of F8 to F13 objective functions and F14 to F23 objective functions are presented in Table 5 and Table 6, respectively, using the proposed CMBO as well as nine compared algorithms. Based on the simulation results, it is determined that the CMBO with high ability to scan the search space is able to converge to quasi-optimal solutions without getting stuck in local optimal points. Therefore, the proposed CMBO has a high capability in the exploration index and is much more competitive than the competing algorithms.

### Execution Time Analysis

In this subsection, studies of the execution time of optimization algorithms in solving objective functions are presented. The experimentation and algorithms are implemented in Matlab R2014a (8.3.0.532) version and run in the environment of Microsoft Windows 10 with 64 bits on Core i-7 processor with 2.40 GHz and 6 GB memory. The average execution time (ave_time) in seconds and the standard deviation for execution time (std_time) are computed as the metrics of performance. To generate and report the results, for each objective function, optimization algorithms utilize 20 independent runs where each run employs 1000 times of iterations.

The results of execution time analysis for all twenty-three objective functions are presented in Table 10. What can be deduced from the simulation results of this analysis is that the proposed CMBO is implemented on optimization problems in less time and has provided quasi-optimal solutions. A comparative review of the CMBO and compared algorithms is presented in Table 11.

## 5. Conclusions and Feature Works

Designed optimization problems in different sciences should be solved using appropriate methods. Optimization algorithms are one of the most widely used and effective methods to provide appropriate solutions to optimization problems. In this paper, a new optimizer called Cat and Mouse-Based Optimizer (CMBO) has been presented that mimics the natural behavior between cats and mice. The mathematical model of the proposed CMBO has been presented based on simulating the cats attack on mice and the escape of mice to shelters. The performance of the CMBO in optimization was tested on a standard set consisting of twenty-three objective functions and the results were compared with the performance of nine algorithms Genetic Algorithm (GA), Particle Swarm Optimization (PSO), Gravitational Search Algorithm (GSA), Teaching-Learning-Based Optimization (TLBO), Grey Wolf Optimizer (GWO), Whale Optimization Algorithm (WOA), Marine Predators Algorithm (MPA), Tunicate Swarm Algorithm (TSA), and Teamwork Optimization Algorithm (TOA). The results of optimization of Unimodal objective functions showed that the proposed CMBO has a high capability in solving this type of optimization problems and has a very good exploitation power. The results of the implementation of the proposed algorithm on the objective functions of high-dimensional and fixed-dimensional multimodal showed the high exploration power of the proposed CMBO in order to accurately scan the search space of optimization problems. Moreover, analyzing the results and comparing the performance of the mentioned algorithms with the performance of the CMBO showed the superiority and more competitiveness of the proposed algorithm.

The conclusions presented in this section about the performance and ability of the proposed CMBO to solve optimization problems were based on the optimization of twenty-three standard objective functions. From a general point of view, in optimization studies, it cannot be claimed that a particular optimization algorithm is the best optimizer to solve all optimization problems. In fact, the algorithm should be used to solve the problems, and based on the results, it should be stated whether the proposed algorithm is generally better than the existing methods or for a set of problems that need to be identified. The important thing about all optimization algorithms is that it is always possible to develop new optimization algorithms that can provide more desirable quasi-optimal solutions that are also closer to the global optimal.

The authors present several ideas as potentials for future studies, including the design of a multi-objective version as well as a binary version of the CMBO. In addition, the application of the proposed CMBO in solving real-life problems and other optimization problems in various sciences is suggestions for further research.

## Figures and Tables

**Table 1:** Proposed well-known optimization algorithms in the recent literature.

Ref.	Algorithm	Main Idea (Inspiration Source)
[16]	Cuckoo Search	Behavior of cuckoo
[17]	Aquila Optimizer	Behavior of Aquila in nature during the process of catching the prey
[18]	Lion Optimization Algorithm	Behavior of lion
[19]	Grasshopper Optimization Algorithm	Grasshopper behavior
[20]	Emperor Penguin Optimizer	The behavior of emperor penguin
[21]	Cat Swarm Optimization Algorithm	Behaviors of cats
[22]	Pity Beetle Algorithm	Aggregation behavior, searching for nest and food
[23]	Mouth Brooding Fish	The behavior of mouthbrooding fish
[24]	Sailfish Optimizer	Group of hunting sailfish
[25]	Following Optimization Algorithm	Relationships between members and the leader of a community
[26]	Multi-Leader Optimizer	The presence of several leaders simultaneously for the population members
[27]	Differential Evolution	the natural phenomenon of evolution
[28]	Evolution Strategy	Darwinian evolution theory
[29]	Biogeography-Based Optimizer	Biogeographic concepts
[30]	Artificial Infectious Disease	SEIQR epidemic model
[31]	Rooted Tree Optimization	Plant roots movement looking for water
[32]	Weighted Superposition Attraction	Weighted superposition of active fields
[33]	Plant Intelligence	Plants nervous system
[34]	Chemotherapy Science	Chemotherapy method
[35]	Tree Growth Algorithm	Trees competition for acquiring light and foods
[36]	Simulated Annealing	Metal annealing process
[37]	Water Cycle Algorithms	Water cycle process and how rivers and streams flow to the sea in the real world
[38]	Water Evaporation Optimization	Evaporation of water molecules
[39]	Galactic Swarm Optimized Motion	The motion of stars, galaxies
[40]	Spring Search Algorithms	Hooke’s law
[41]	Collective Decision Optimization	The social behavior of human beings
[42]	Very Optimistic Method	Real-life practices of successful persons
[43]	Momentum Search Algorithm	Momentum law and Newton’s laws of motion
[44]	Archimedes Optimization Algorithm	Law of physics Archimedes’ Principle which imitates the principle of buoyant force exerted upward on an object
[45]	Dice Game Optimizer	Rules governing the game of dice and the impact of players on each other
[46]	Orientation Search Algorithm	Game of orientation, in which players move in the direction of a referee
[47]	Hide Objects Game Optimization	Behavior and movements of players to find a hidden object
[48]	Football Game Based Optimization	Simulation of behavior of clubs in football league.
[49]	Darts Game Optimizer	Rules of the Darts game
[50]	Shell Game Optimization	Rules of the shell game

**Table 2:** The various steps of the proposed CMBO for the first iteration in sphere function solving.

	Step 1		Step 2	Step 3			Step 4		Step 5			Step 6		
	*X*		*F(X)*	*X^S^*		*F^S^(X)*	Cats	Mice	*C*		*F^C^*	*M*		*F^m^*
	*x_1_*	*x_2_*		$x_{1}^{S}$	$x_{2}^{S}$				*c*_1_	*c*_2_		*m*_1_	*m*_2_	
*X*_1_	69.00641	−74.5553	10,320.38	−36.7889	19.51363	1734.208		*M*_1_				−36.7889	19.51363	1734.208
*X*_2_	18.96709	−45.9773	2473.659	18.96709	−45.9773	2473.659		*M*_2_				18.96709	−45.9773	2473.659
*X*_3_	99.35621	−34.7797	11,081.28	46.22603	18.41816	2476.074		*M*_3_				−12.7845	3.444065	175.3053
*X*_4_	−36.7889	19.51363	1734.208	41.24409	−45.8968	3807.588		*M*_4_				32.03547	−45.8968	3132.784
*X*_5_	46.22603	18.41816	2476.074	68.51469	−19.8287	5087.439		*M*_5_				−3.47199	−20.895	448.6563
*X*_6_	−91.4795	76.26784	14,185.29	57.48351	58.62186	6740.877	*C*_1_		52.12189	−42.9835	4564.272			
*X*_7_	68.51469	−19.8287	5087.439	69.00641	−74.5553	10,320.38	*C*_2_		−24.7895	−40.9822	2294.059			
*X*_8_	−64.1203	−80.2158	10,545.99	−64.1203	−80.2158	10,545.99	*C*_3_		−51.4096	−50.6034	5203.653			
*X*_9_	41.24409	−45.8968	3807.588	99.35621	−34.7797	11,081.28	*C*_4_		87.51208	−30.541	8591.116			
*X*_10_	57.48351	58.62186	6740.877	−91.4795	76.26784	14,185.29	*C*_5_		−16.7554	52.75574	3063.913			
Full implementation														
Best Solution: *x*_1_ = 3.51 × 10^−12^, *x*_2_ = 6.73 × 10^−12^ and *F(X)* = 5.7626 × 10^−23^														

**Table 3:** Parameter values for the compared algorithms.

Algorithm	Parameter	Value
GA		
	Type	Real coded
	Selection	Roulette wheel (Proportionate)
	Crossover	Whole arithmetic (Probability = 0.8, $\alpha \in \left[-0.5,\;1.5\right]$)
	Mutation	Gaussian (Probability = 0.05)
PSO		
	Topology	Fully connected
	Cognitive and social constant	(*C*_1_, *C*_2_) = (2, 2)
	Inertia weight	Linear reduction from 0.9 to 0.1
	Velocity limit	10% of dimension range
GSA		
	Alpha, *G*_0_, *R_norm_*, *R_power_*	20, 100, 2, 1
TLBO		
	*T_F_*: teaching factor	$T_{F}=$ round $\left[\left(1+rand\right)\right]$
	random number	*rand* is a random number in the range $\left[0-1\right].$
GWO		
	Convergence parameter (*a*)	*a*: Linear reduction from 2 to 0.
WOA		
	Convergence parameter (*a*)	*a*: Linear reduction from 2 to 0.
	*r* is a random vector in $\left[0,1\right].$	
	*l* is a random number in $\left[-1,1\right].$	
TSA		
	P_min_ and P_max_	1, 4
	*C*_1_, *C*_2_, *C*_3_	random numbers, which lie in the range $\left[0-1\right].$
MPA		
	Constant number	*p* = 0.5
	Random vector	*R* is a vector of uniform random numbers in the range $\left[0-1\right].$
	Fish Aggregating Devices (*FADs*)	*FADs*= 0.2
	Binary vector	*U*= 0 or 1
TOA		
	Update index	I= round $\left[\left(1+rand\right)\right]$
	*r*	*r* is a uniform random number in the range $\left[0-1\right].$

**Table 4:** Optimization results of CMBO and other algorithms on unimodal function.

		CMBO	TOA	MPA	TSA	WOA	GWO	TLBO	GSA	PSO	GA
F_1_	ave	2.69 × 10^−236^	**0**	3.2715 × 10^−21^	7.71 × 10^−38^	2.1741 × 10^−9^	1.09 × 10^−58^	8.3373 × 10^−60^	2.0255 × 10^−17^	1.7740 × 10^−5^	13.2405
	std	0	**0**	4.6153 × 10^−21^	7.00 × 10^−21^	7.3985 × 10^−25^	5.1413 × 10^−74^	4.9436 × 10^−76^	1.1369 × 10^−32^	6.4396 × 10^−21^	4.7664 × 10^−15^
F_2_	ave	6.88 × 10^−121^	**0**	1.57 × 10^−12^	8.48 × 10^−39^	0.5462	1.2952 × 10^−34^	7.1704 × 10^−35^	2.3702 × 10^−8^	0.3411	2.4794
	std	2.46 × 10^−135^	**0**	1.42 × 10^−12^	5.92 × 10^−41^	1.7377 × 10^−16^	1.9127 × 10^−50^	6.6936 × 10^−50^	5.1789 × 10^−24^	7.4476 × 10^−17^	2.2342 × 10^−15^
F_3_	ave	2.44 × 10^−60^	**0**	0.0864	1.15 × 10^−21^	1.7634 × 10^−8^	7.4091 × 10^−15^	2.7531 × 10^−15^	279.3439	589.492	1536.8963
	std	1.82 × 10^−67^	**0**	0.1444	6.70 × 10^−21^	1.0357 × 10^−23^	5.6446 × 10^−30^	2.6459 × 10^−31^	1.2075 × 10^−13^	7.1179 × 10^−13^	6.6095 × 10^−13^
F_4_	ave	1.04 × 10^−93^	**0**	2.6 × 10^−8^	1.33 × 10^−23^	2.9009 × 10^−5^	1.2599 × 10^−14^	9.4199 × 10^−15^	3.2547 × 10^−9^	3.9634	2.0942
	std	2.09 × 10^−108^	**0**	9.25 × 10^−9^	1.15 × 10^−22^	1.2121 × 10^−20^	1.0583 × 10^−29^	2.1167 × 10^−30^	2.0346 × 10^−24^	1.9860 × 10^−16^	2.2342 × 10^−15^
F_5_	ave	**24.87011**	26.2476	46.049	28.8615	41.7767	26.8607	146.4564	36.10695	50.26245	310.4273
	std	**1.91 × 10^−14^**	3.26 × 10^−14^	0.4219	4.76 × 10^−3^	2.5421 × 10^−14^	0	1.9065 × 10^−14^	3.0982 × 10^−14^	1.5888 × 10^−14^	2.0972 × 10^−13^
F_6_	ave	**0**	**0**	0.398	7.10 × 10^−21^	1.6085 × 10^−9^	0.6423	0.4435	0	20.25	14.55
	std	**0**	**0**	0.1914	1.12 × 10^−25^	4.6240 × 10^−25^	6.2063 × 10^−17^	4.2203 × 10^−16^	0	1.2564	3.1776 × 10^−15^
F_7_	ave	0.002709	**9.92 × 10^−06^**	0.0018	3.72 × 10^−4^	0.0205	0.0008	0.0017	0.0206	0.1134	5.6799 × 10^−3^
	std	1.94 × 10^−19^	**1.74 × 10^−20^**	0.001	5.09 × 10^−5^	1.5515 × 10^−18^	7.2730 × 10^−20^	3.87896 × 10^−19^	2.7152 × 10^−18^	4.3444 × 10^−17^	7.7579 × 10^−19^

**Table 5:** Optimization results of CMBO and other algorithms on high-dimensional function.

		CMBO	TOA	MPA	TSA	WOA	GWO	TLBO	GSA	PSO	GA
F_8_	ave	−6561.15	**−9631.41**	−3594.1632	−5740.3388	−1663.9782	−5885.1172	−7408.6107	−2849.0724	−6908.6558	−8184.4142
	std	**1.83 × 10^−12^**	3.86 × 10^−12^	811.32651	41.5	716.3492	467.5138	513.5784	264.3516	625.6248	833.2165
F_9_	ave	**0**	**0**	140.1238	5.70 × 10^−3^	4.2011	8.5265 × 10^−15^	10.2485	16.2675	57.0613	62.4114
	std	**0**	**0**	26.3124	1.46 × 10^−3^	4.3692 × 10^−15^	5.6446 × 10^−30^	5.5608 × 10^−15^	3.1776 × 10^−15^	6.3552 × 10^−15^	2.5421 × 10^−14^
F_10_	ave	4.44 × 10^−15^	**8.88 × 10^−16^**	9.6987 × 10^−12^	9.80 × 10^−14^	0.3293	1.7053 × 10^−14^	0.2757	3.5673 × 10^−9^	2.1546	3.2218
	std	**0**	**0**	6.1325 × 10^−12^	4.51 × 10^−12^	1.9860 × 10^−16^	2.7517 × 10^−29^	2.5641 × 10^−15^	3.6992 × 10^−25^	7.9441 × 10^−16^	5.1636 × 10^−15^
F_11_	ave	**0**	**0**	**0**	1.00 × 10^−7^	0.1189	0.0037	0.6082	3.7375	0.0462	1.2302
	std	**0**	**0**	**0**	7.46 × 10^−7^	8.9991 × 10^−17^	1.2606 × 10^−18^	1.9860 × 10^−16^	2.7804 × 10^−15^	3.1031 × 10^−18^	8.4406 × 10^−16^
F_12_	ave	**1.10 × 10^−08^**	0.2463	0.0851	0.0368	1.7414	0.0372	0.0203	0.0362	0.4806	0.047
	std	**1.66 × 10^−22^**	7.45 × 10^−17^	0.0052	1.5461 × 10^−2^	8.1347 × 10^−12^	4.3444 × 10^−17^	7.7579 × 10^−19^	6.2063 × 10^−18^	1.8619 × 10^−16^	4.6547 × 10^−18^
F_13_	ave	**1.78 × 10^−07^**	1.25	0.4901	2.9575	0.3456	0.5763	0.3293	0.002	0.5084	1.2085
	std	**3.10 × 10^−18^**	4.47 × 10^−16^	0.1932	1.5682 × 10^−12^	3.25391 × 10^−12^	2.4825 × 10^−15^	2.1101 × 10^−14^	4.2617 × 10^−14^	4.9650 × 10^−17^	3.2272 × 10^−16^

**Table 6:** Optimization results of CMBO and other algorithms on fixed-dimensional function.

		CMBO	TOA	MPA	TSA	WOA	GWO	TLBO	GSA	PSO	GA
F_14_	ave	**0.998**	0.9980	0.998	1.9923	0.998	3.7408	2.2721	3.5913	2.1735	0.9986
	std	**0**	4.72 × 10^−16^	4.2735 × 10^−16^	2.6548 × 10^−7^	9.4336 × 10^−16^	6.4545 × 10^−15^	1.9860 × 10^−16^	7.9441 × 10^−16^	7.9441 × 10^−16^	1.5640 × 10^−15^
F_15_	ave	**0.000307**	0.000307	0.003	0.0004	0.0049	0.0063	0.0033	0.0024	0.0535	5.3952 × 10^−2^
	std	**1.21 × 10^−20^**	1.16 × 10^−18^	4.0951 × 10^−15^	9.0125 × 10^−4^	3.4910 × 10^−18^	1.1636 × 10^−18^	1.2218 × 10^−17^	2.9092 × 10^−19^	3.8789 × 10^−19^	7.0791 × 10^−18^
F_16_	ave	**−1.03163**	−1.0316	−1.0316	−1.0316	−1.0316	−1.0316	−1.0316	−1.0316	−1.0316	−1.0316
	std	**1.47 × 10^−16^**	1.99 × 10^−16^	4.4652 × 10^−16^	2.6514 × 10^−16^	9.9301 × 10^−16^	3.9720 × 10^−16^	1.4398 × 10^−15^	5.9580 × 10^−16^	3.4755 × 10^−16^	7.9441 × 10^−16^
F_17_	ave	**0.3978**	0.3978	0.3979	0.3991	0.4047	0.3978	0.3978	0.3978	0.7854	0.4369
	std	**0**	9.93 × 10^−17^	9.1235 × 10^−15^	2.1596 × 10^−16^	2.4825 × 10^−17^	8.6888 × 10^−17^	7.4476 × 10^−17^	9.9301 × 10^−17^	4.9650 × 10^−17^	4.9650 × 10^−17^
F_18_	ave	**3**	**3**	3	3	3	3	3.0009	3	3	4.3592
	std	**0**	**0**	1.9584 × 10^−15^	2.6528 × 10^−15^	5.6984 × 10^−15^	2.0853 × 10^−15^	1.5888 × 10^−15^	6.9511 × 10^−16^	3.6741 × 10^−15^	5.9580 × 10^−16^
F_19_	ave	**−3.86278**	−3.86278	−3.8627	−3.8066	−3.8627	−3.8621	−3.8609	−3.8627	−3.8627	−3.85434
	std	**1.83 × 10^−16^**	2.68 × 10^−16^	4.2428 × 10^−15^	2.6357 × 10^−15^	3.1916 × 10^−15^	2.4825 × 10^−15^	7.3483 × 10^−15^	8.3413 × 10^−15^	8.9371 × 10^−15^	9.9301 × 10^−17^
F_20_	ave	**−3.322**	−3.322	−3.3211	−3.3206	−3.2424	−3.2523	−3.2014	−3.0396	−3.2619	−2.8239
	std	**1.59 × 10^−16^**	1.69 × 10^−15^	1.1421 × 10^−11^	5.6918 × 10^−15^	7.9441 × 10^−16^	2.1846 × 10^−15^	1.7874 × 10^−15^	2.1846 × 10^−14^	2.9790 × 10^−16^	3.97205 × 10^−16^
F_21_	ave	**−10.1532**	−10.1532	−10.1532	−5.5021	−7.4016	−9.6452	−9.1746	−5.1486	−5.3891	−4.3040
	std	**1.15 × 10^−16^**	1.39 × 10^−15^	2.5361 × 10^−11^	5.4615 × 10^−13^	2.3819 × 10^−11^	6.5538 × 10^−15^	8.5399 × 10^−15^	2.9790 × 10^−16^	1.4895 × 10^−15^	1.5888 × 10^−15^
F_22_	ave	**−10.4029**	−10.4029	−10.4029	−5.0625	−8.8165	−10.4025	−10.0389	−9.0239	−7.6323	−5.1174
	std	**1.39 × 10^−16^**	3.18 × 10^−15^	2.8154 × 10^−11^	8.4637 × 10^−14^	6.7524 × 10^−15^	1.9860 × 10^−15^	1.5292 × 10^−14^	1.6484 × 10^−12^	1.5888 × 10^−15^	1.2909 × 10^−15^
F_23_	ave	**−10.5364**	−10.5364	−10.5364	−10.3613	−10.0003	−10.1302	−9.2905	−8.9045	−6.1648	−6.5621
	std	**1.35 × 10^−16^**	7.94 × 10^−16^	3.9861 × 10^−11^	7.6492 × 10^−12^	9.1357 × 10^−15^	4.5678 × 10^−15^	1.1916 × 10^−15^	7.1497 × 10^−14^	2.7804 × 10^−15^	3.8727 × 10^−15^

**Table 7:** Statistical analysis results from the Wilcoxon test (*p* ≥ 0.05).

Compared Algorithms	Unimodal	High-Dimensional Multi Modal	Fixed-Dimensional Multi Modal
CMBO vs. TOA	0.4375	0.4375	0.625
CMBO vs. TSA	0.109375	0.0625	0.0625
CMBO vs. MPA	0.015625	0.03125	0.003906
CMBO vs. WOA	0.015625	0.03125	0.007813
CMBO vs. GWO	0.15625	0.03125	0.011719
CMBO vs. TLBO	0.15625	0.4375	0.005859
CMBO vs. GSA	0.03125	0.03125	0.019531
CMBO vs. PSO	0.015625	0.4375	0.003906
CMBO vs. GA	0.015625	0.4375	0.001953

**Table 8:** Results of the algorithm sensitivity analysis to the number of population members.

Objective Functions	Number of Population Members			
	20	30	50	80
F_1_	3.7 × 10^−201^	1.4 × 10^−214^	2.7 × 10^−236^	1.1 × 10^−243^
F_2_	5.4 × 10^−137^	1.4 × 10^−126^	6.9 × 10^−121^	4.3 × 10^−119^
F_3_	8.69 × 10^−74^	8.34 × 10^−60^	2.44 × 10^−60^	2.42 × 10^−57^
F_4_	5.5 × 10^−108^	1.23 × 10^−98^	1.04 × 10^−93^	4.96 × 10^−92^
F_5_	26.86162	25.87908	24.87011	24.58636
F_6_	0	0	0	0
F_7_	0.008517	0.006639	0.002709	0.001691
F_8_	−4696	−7900.45	−6561.15	−7142.03
F_9_	0	0	0	0
F_10_	4.44 × 10^−15^	4.44 × 10^−15^	4.44 × 10^−15^	4.44 × 10^−15^
F_11_	0	0	0	0
F_12_	0.038614	0.003171	1.1 × 10^−08^	1.36 × 10^−09^
F_13_	1.281798	0.305144	1.78 × 10^−07^	5.22 × 10^−09^
F_14_	1.593234	1.196414	0.998	0.998004
F_15_	0.000418	0.000311	0.000307	0.000307
F_16_	−1.03163	−1.03163	−1.03163	−1.03163
F_17_	0.397887	0.397887	0.3978	0.397887
F_18_	9.75	3	3	3
F_19_	−3.82413	−3.86278	−3.86278	−3.86278
F_20_	−3.3005	−3.30416	−3.322	−3.322
F_21_	−8.71417	−8.61749	−10.1532	−10.1532
F_22_	−7.84302	−8.35605	−10.4029	−10.4029
F_23_	−8.57191	−9.61404	−10.5364	−10.5364

**Table 9:** Results of the algorithm sensitivity analysis to the maximum number of iterations.

Objective Functions	Maximum number of iterations			
	100	500	800	1000
F_1_	9.54 × 10^−20^	6.7 × 10^−115^	9.3 × 10^−187^	2.7 × 10^−236^
F_2_	9.48 × 10^−11^	3.22 × 10^−59^	1.47 × 10^−95^	6.9 × 10^−121^
F_3_	0.047734	7.69 × 10^−24^	7.75 × 10^−41^	2.44 × 10^−60^
F_4_	5.41 × 10^−08^	1.27 × 10^−45^	8.96 × 10^−74^	1.04 × 10^−93^
F_5_	27.78238	26.04638	25.62131	24.87011
F_6_	0	0	0	0
F_7_	0.012014	0.005967	0.005116	0.002709
F_8_	−3642.94	−4496.48	−5014.61	−6561.15
F_9_	0	0	0	0
F_10_	7.19 × 10^−11^	4.44 × 10^−15^	4.44 × 10^−15^	4.44 × 10^−15^
F_11_	0	0	0	0
F_12_	0.023937	0.000129	1.23 × 10^−05^	1.1 × 10^−08^
F_13_	0.324195	0.030145	0.016247	1.78 × 10^−07^
F_14_	1.096872	0.998004	0.998004	0.998
F_15_	0.000529	0.000341	0.000308	0.000307
F_16_	−1.03163	−1.03163	−1.03163	−1.03163
F_17_	0.397887	0.397887	0.397887	0.3978
F_18_	3	3	3	3
F_19_	−3.86278	−3.86278	−3.86278	−3.86278
F_20_	−3.31584	−3.32199	−3.322	−3.322
F_21_	−8.93555	−9.64077	−10.1532	−10.1532
F_22_	−9.13251	−10.4027	−10.4029	−10.4029
F_23_	−10.4926	−10.5364	−10.5362	−10.5364

**Table 10:** Comparison of average execution time (ave_time) in seconds and the standard deviation for execution time (std_time).

		CMBO	TOA	MPA	TSA	WOA	GWO	TLBO	GSA	PSO	GA
F_1_	ave_time	2.06327	2.288047	2.828934	2.27456	2.856446	3.142379	3.662429	9.024897	3.685416	3.876233
	std_time	0.008802	0.103601	0.021766	0.00882	0.039718	0.01458	0.008365	0.110843	0.01622	0.044052
F_2_	ave_time	2.149418	2.33419	2.222183	2.496359	3.143495	3.000322	3.747463	9.602311	3.425936	3.34707
	std_time	0.009976	0.05675	0.00619	0.004159	0.012551	0.001226	0.00205	0.029098	0.001869	0.001112
F_3_	ave_time	3.759693	6.014573	6.474818	5.805883	10.96679	6.132736	13.31042	11.65223	14.68167	12.16981
	std_time	0.049343	0.184878	0.056553	0.003913	0.020291	0.002126	0.005486	0.05522	0.023614	0.063015
F_4_	ave_time	2.095707	2.303496	2.830211	2.429061	2.82791	2.884421	3.475663	9.399065	3.298336	3.218456
	std_time	0.000425	0.058168	0.006382	0.001313	0.002522	0.000774	0.000146	0.095684	0.002026	0.001128
F_5_	ave_time	2.319993	2.668194	3.271111	2.837473	4.076267	3.362668	4.743216	9.85618	4.510391	4.74454
	std_time	0.001194	0.098008	0.020855	0.001889	0.004884	0.0012	0.013286	0.000635	0.004473	0.015477
F_6_	ave_time	2.051842	2.142897	2.778443	2.388851	2.636699	2.884786	3.543828	9.739783	2.826627	3.964025
	std_time	0.000734	0.053895	0.002564	0.000531	0.000103	0.000545	0.003082	0.081756	0.00079	0.002177
F_7_	ave_time	2.799308	3.426696	5.361778	4.208485	7.73167	4.624996	9.5011	11.0389	10.25112	8.948355
	std_time	0.000298	0.138022	0.010684	0.001381	0.013762	0.000748	0.01131	0.002795	0.062275	0.003807
F_8_	ave_time	2.368102	2.787053	3.54212	3.038116	4.470513	3.45166	5.934958	10.15033	6.189501	5.35854
	std_time	0.001774	0.146471	0.028588	0.001872	0.008998	0.000947	0.024404	0.002614	0.027143	0.006958
F_9_	ave_time	2.105224	2.141418	3.207098	2.70311	2.998242	2.962939	5.075711	10.07901	4.697519	4.632448
	std_time	0.000457	0.022495	0.00947	0.000574	0.001451	0.001075	0.007871	0.034299	0.006383	0.001877
F_10_	ave_time	2.119633	2.213827	3.199752	2.685157	3.250468	2.951537	4.663569	9.862325	4.465213	4.851987
	std_time	0.000675	0.106657	0.008234	0.001502	0.002534	0.000494	0.002845	0.030374	0.003311	0.000453
F_11_	ave_time	2.382237	2.702994	3.627042	3.029926	4.341168	3.544234	5.783952	10.09065	5.962496	5.935665
	std_time	0.000521	0.090901	0.002333	0.006447	0.004761	0.011232	0.004814	0.001281	0.004878	0.010718
F_12_	ave_time	4.689757	6.401408	9.786492	7.746796	16.08114	8.081277	22.27949	13.02264	23.24494	18.18917
	std_time	0.000582	0.136896	0.121341	0.003636	0.010787	0.005813	0.145335	0.001683	0.143479	0.016131
F_13_	ave_time	4.683313	6.317049	9.715068	7.882488	16.03426	8.216492	21.93277	12.87604	22.93827	17.26237
	std_time	0.00164	0.142056	0.059173	0.015565	0.009175	0.040121	0.238577	0.080775	0.040952	0.026452
F_14_	ave_time	6.77659	11.05703	10.17388	11.25044	28.35404	10.67249	38.58243	10.43203	42.03153	31.15071
	std_time	0.024394	0.495939	0.132818	0.007729	0.043197	0.004033	0.463309	0.00956	0.793664	0.187898
F_15_	ave_time	1.162517	2.036434	1.674953	1.143977	2.66252	1.168071	4.228257	4.381512	2.407291	3.348114
	std_time	0.0006	0.076443	0.004268	0.001227	0.003729	0.000474	0.004635	0.000891	0.003895	0.000252
F_16_	ave_time	0.981154	1.965747	1.553408	1.014295	2.554895	0.98797	4.155264	3.895143	2.183457	3.277226
	std_time	0.000487	0.097936	0.013704	0.001489	0.002058	0.000326	0.002632	0.006833	0.000941	0.000117
F_17_	ave_time	0.976854	1.818405	1.235874	0.86938	2.127932	0.865444	3.596898	3.391429	1.754779	2.926444
	std_time	0.025755	0.076691	0.005655	0.000666	0.001913	0.000584	0.005077	0.002162	0.001703	0.000751
F_18_	ave_time	0.895712	1.828383	1.161598	0.778106	1.943841	0.827821	3.474254	3.32534	1.553584	3.013364
	std_time	0.000171	0.115063	0.000996	5.56E-05	0.000193	0.000381	0.004198	0.007157	0.002831	0.002876
F_19_	ave_time	1.15748	2.291343	1.500319	1.165046	2.852	1.19711	4.232431	3.811885	2.8576	3.788615
	std_time	0.002134	0.265954	0.00141	0.000364	0.00364	0.000711	0.007588	0.009134	0.009284	0.000403
F_20_	ave_time	1.282607	2.124999	1.57968	1.384633	3.174224	1.359368	4.217968	4.155625	3.802493	4.105984
	std_time	0.000401	0.068302	0.001507	0.001233	0.004248	0.000464	0.003929	0.00207	0.082165	0.003145
F_21_	ave_time	1.325595	2.387586	1.958585	1.625469	3.975843	1.81357	5.617517	3.935374	3.834105	7.570488
	std_time	0.001363	0.086822	0.021254	0.002127	0.072322	0.009181	0.026092	0.004605	0.006494	8.978715
F_22_	ave_time	1.460515	2.645545	2.197999	1.762546	4.159347	1.714076	6.661208	4.227989	4.859716	5.190246
	std_time	0.004856	0.142053	0.018205	0.00058	0.002778	0.000859	0.005957	0.03825	0.028151	0.001234
F_23_	ave_time	1.558004	2.857437	2.69353	2.168531	5.108969	2.032143	7.707782	5.007117	6.206333	6.102901
	std_time	0.001463	0.159845	0.019916	0.002441	0.007591	0.003044	0.00225	0.005821	0.15752	0.001592

**Table 11:** Comparative review for CMBO and compared algorithms.

Algorithm	Disadvantage	Advantage
GA	High memory consumption, having control parameters, and poor local search.	Good global search, simplicity and comprehensibility
PSO	Having control parameters, poor convergence and entrapment in local optimum areas.	Simplicity of the relationship and its implementation.
GSA	High computations, time consuming, having several control parameters, and poor convergence in complex objective functions.	Easy implementation, fast convergence in simple problems, and low computational cost.
TLBO	Poor convergence rate.	Good global search, simplicity, and not requiring any parameter.
GWO	Low convergence speed, poor local search, and low accuracy in solving complex problems.	Fast convergence due to continuous reduction of search space, less storage and computational requirements, and easy to implement due to its simple structure.
WOA	Low accuracy, slow convergence, and easy to fall into local optimum.	Simple structure, less required operator, and having appropriate balance between exploration and exploitation.
MPA	High computations, time consuming, and having control parameters.	Good global search and fast convergence.
TSA	Poor convergence, having control parameters and fall to local optimal solutions in solving high-dimensional multimodal problems.	Fast convergence, good global search, having appropriate balance between exploration and exploitation.
TOA	Fall to local optimal solutions in solving high-dimensional multimodal problems.	Not requiring any parameter, good global search, having appropriate balance between exploration and exploitation, and fast convergence.
CMBO	The important thing about all optimization algorithms is that it cannot be claimed that one particular algorithm is the best optimizer for all optimization problems. It is also always possible to develop new optimization algorithms that can provide more desirable quasi-optimal solutions that are also closer to the global optimal.	Easy implementation, simplicity of equations, lack of control parameters, proper exploitation, proper exploration, high convergence power, and not getting caught up in local optimal solutions.

## Data Availability

The data present in this study are available on request from the author M.D.

## Appendix A

Complete information and details on the standard objective functions of unimodal, high-dimensional multimodal, and fixed-dimensional multimodal are provided in Table A1, Table A2 and Table A3, respectively.

**Table A1:** Unimodal test functions.

Objective Function	Range	Dimensions	F_min_
$F_{1}\left(x\right)=\sum_{i=1}^{m}x_{i}^{2}$	$\left[-100,100\right]$	30	0
$F_{2}\left(x\right)=\sum_{i=1}^{m}\left|x_{i}\right|+\prod_{i=1}^{m}\left|x_{i}\right|$	$\left[-10,10\right]$	30	0
$F_{3}\left(x\right)=\sum_{i=1}^{m}{\left(\sum_{j=1}^{i}x_{i}\right)}^{2}$	$\left[-100,100\right]$	30	0
$F_{4}\left(x\right)=max\left\{\left|x_{i}\right|\;,\;1\leq i\leq m\right\}$	$\left[-100,100\right]$	30	0
$F_{5}\left(x\right)=\sum_{i=1}^{m-1}\left[100{\left(x_{i+1}-x_{i}^{2}\right)}^{2}+{\left(x_{i}-1\right)}^{2})\right]$	$\left[-30,30\right]$	30	0
$F_{6}\left(x\right)=\sum_{i=1}^{m}{\left(\left[x_{i}+0.5\right]\right)}^{2}$	$\left[-100,100\right]$	30	0
$F_{7}\left(x\right)=\sum_{i=1}^{m}ix_{i}^{4}+random\left(0,1\right)$	$\left[-1.28,1.28\right]$	30	0

**Table A2:** High-dimensional multimodal test functions.

Objective Function	Range	Dimensions	F_min_
$F_{8}\left(x\right)=\sum_{i=1}^{m}-x_{i}\;\sin\left(\sqrt{\left|x_{i}\right|}\right)$	$\left[-500,500\right]$	30	−12,569
$F_{9}\left(x\right)=\sum_{i=1}^{m}\left[\;x_{i}^{2}-10\cos\left(2\pi x_{i}\right)+10\right]$	$\left[-5.12,5.12\right]$	30	0
$F_{10}\left(x\right)=-20\exp\left(-0.2\sqrt{\frac{1}{m}\sum_{i=1}^{m}x_{i}^{2}}\right)-\exp\left(\frac{1}{m}\sum_{i=1}^{m}\cos\left(2\pi x_{i}\right)\right)+20+e$	$\left[-32,32\right]$	30	0
$F_{11}\left(x\right)=\frac{1}{4000}\sum_{i=1}^{m}x_{i}^{2}-\prod_{i=1}^{m}cos\left(\frac{x_{i}}{\sqrt{i}}\right)+1$	$\left[-600,600\right]$	30	0
$\begin{array}{cc} F_{12}\left(x\right)=\frac{\pi }{m}\;\{10\sin & \left(\pi y_{1}\right)\\ & +\sum_{i=1}^{m}{\left(y_{i}-1\right)}^{2}\left[1+10\sin^{2}\left(\pi y_{i+1}\right)\right]+{\left(y_{n}-1\right)}^{2}\}\\ & +\sum_{i=1}^{m}u\left(x_{i},10,100,4\right) \end{array}$ $u\left(x_{i},a,i,n\right)=\left\{\begin{array}{c} k{\left(x_{i}-a\right)}^{n},\;x_{i}>-a;\\ 0,\;-a<x_{i}<a;\\ k{\left(-x_{i}-a\right)}^{n},\;x_{i}<-a. \end{array}\right.$	$\left[-50,50\right]$	30	0
$\begin{array}{cc} F_{13}\left(x\right)=0.1\{\;\sin^{2} & \left(3\pi x_{1}\right)\\ & +\sum_{i=1}^{m}{\left(x_{i}-1\right)}^{2}[1+\sin^{2}\left(3\pi x_{i}+1\right)]+{\left(x_{n}-1\right)}^{2}[1\\ & +\sin^{2}\left(2\pi x_{m}\right)]\}+\sum_{i=1}^{m}u\left(x_{i},5,100,4\right) \end{array}$	$\left[-50,50\right]$	30	0

**Table A3:** Fixed-dimensional multimodal test functions.

Objective Function	Range	Dimensions	F_min_
$F_{14}\left(x\right)={\left(\frac{1}{500}+\sum_{j=1}^{25}\frac{1}{j+\sum_{i=1}^{2}{\left(x_{i}-a_{ij}\right)}^{6}}\right)}^{-1}$	$\left[-65.53,65.53\right]$	2	0.998
$F_{15}\left(x\right)=\sum_{i=1}^{11}{\left[a_{i}-\frac{x_{1}\left(b_{i}^{2}+b_{i}x_{2}\right)}{b_{i}^{2}+b_{i}x_{3}+x_{4}}\right]}^{2}$	$\left[-5,5\right]$	4	0.00030
$F_{16}\left(x\right)=4x_{1}^{2}-2.1x_{1}^{4}+\frac{1}{3}x_{1}^{6}+x_{1}x_{2}-4x_{2}^{2}+4x_{2}^{4}$	$\left[-5,5\right]$	2	−1.0316
$F_{17}\left(x\right)={\left(x_{2}-\frac{5.1}{4\pi^{2}}x_{1}^{2}+\frac{5}{\pi }x_{1}-6\right)}^{2}+10\left(1-\frac{1}{8\pi }\right)cosx_{1}+10$	[−5,10]×[0,15]	2	0.398
$\begin{array}{cc} F_{18}\left(x\right)=[1+(x_{1} & +x_{2}+1){}^{2}(19-14x_{1}+3x_{1}^{2}-14x_{2}+6x_{1}x_{2}\\ & +3x_{2}^{2})]\times [30+{\left(2x_{1}-3x_{2}\right)}^{2}\times (18-32x_{1}+12x_{1}^{2}\\ & +48x_{2}-36x_{1}x_{2}+27x_{2}^{2})] \end{array}$	$\left[-5,5\right]$	2	3
$F_{19}\left(x\right)=-\sum_{i=1}^{4}c_{i}\exp\left(-\sum_{j=1}^{3}a_{ij}{\left(x_{j}-P_{ij}\right)}^{2}\right)$	$\left[0,1\right]$	3	−3.86
$F_{20}\left(x\right)=-\sum_{i=1}^{4}c_{i}\exp\left(-\sum_{j=1}^{6}a_{ij}{\left(x_{j}-P_{ij}\right)}^{2}\right)$	$\left[0,1\right]$	6	−3.22
$F_{21}\left(x\right)=-\sum_{i=1}^{5}{\left[\left(X-a_{i}\right){\left(X-a_{i}\right)}^{T}+6c_{i}\right]}^{-1}$	$\left[0,10\right]$	4	−10.1532
$F_{22}\left(x\right)=-\sum_{i=1}^{7}{\left[\left(X-a_{i}\right){\left(X-a_{i}\right)}^{T}+6c_{i}\right]}^{-1}$	$\left[0,10\right]$	4	−10.4029
$F_{23}\left(x\right)=-\sum_{i=1}^{10}{\left[\left(X-a_{i}\right){\left(X-a_{i}\right)}^{T}+6c_{i}\right]}^{-1}$	$\left[0,10\right]$	4	−10.5364
